# Unraveling the Missing Pieces: Exploring the Gaps in Understanding Chagas Cardiomyopathy

**DOI:** 10.7759/cureus.66955

**Published:** 2024-08-15

**Authors:** Keerthana Veluswami, Sudipta Rao, Shailesh Aggarwal, Sweatha Mani, Abirami Balasubramanian

**Affiliations:** 1 Internal Medicine, Stanley Medical College, Chennai, IND; 2 Internal Medicine, JSS Medical College, Mysore, IND; 3 Internal Medicine, K.A.P. Viswanatham Government Medical College, Tiruchirappalli, IND

**Keywords:** benznidazole, nifurtimox, right bundle branch block, left anterior fascicular block, sudden cardiac death (scd), trypanosoma cruzi, chronic chagas disease, chagas cardiomyopathy

## Abstract

Chagas cardiomyopathy affects a considerable number of patients infected with the protozoan *Trypanosoma cruzi (T. cruzi) *and remains one of the most neglected tropical diseases despite being a significant contributor to morbidity and mortality in both endemic regions of Latin America and non-endemic countries like the United States. Since its discovery almost a century ago, knowledge gaps still exist in the mechanisms involved in the pathogenesis of Chagas cardiomyopathy, and numerous challenges exist in its diagnosis and treatment. This article reviews the main pathogenetic mechanisms involved in the progression of Chagas cardiomyopathy, which has been proposed as a result of years of research. It also emphasizes the challenges involved in the diagnosis of the asymptomatic indeterminate phase and has focused on several diagnostic techniques, including echocardiography, electrocardiogram (ECG), magnetic resonance imaging (MRI), and nuclear imaging in diagnosing symptomatic Chagas cardiomyopathy. In this article, we have also provided a brief overview of the current treatment of Chagas cardiomyopathy, which is not etiology-specific but instead derived from the knowledge acquired from the treatment of other cardiomyopathies.

## Introduction and background

Chagas disease, a neglected tropical disease, also referred to as American trypanosomiasis, caused by the protozoan parasite *Trypanosoma cruzi* (*T. cruzi*), affects multiple systems (cardiovascular, digestive, and central nervous systems) [1, 2]. A physician from Brazil, Carlos Chagas (1879-1934), discovered the Chagas disease in 1909 [1]. Approximately, *T. cruzi* has been estimated to have infected approximately six to seven million people worldwide, predominantly in Latin America, with an additional 75 million people believed to be at risk of contracting the infection [3]. Around 20%-40% of the infected people are affected with Chagas cardiomyopathy, the cardiac form of Chagas disease, and it is the most common cause of non-ischemic cardiomyopathy in Latin America [4]. Owing to migration, Chagas disease, originally confined to the rural areas of Latin America, is currently emerging as an increasing public health issue in the urban areas of Latin America and is extending to regions beyond the borders of Latin America like Spain, Canada, Australia, and the United States [5].

The endemic *T. cruzi* transmission among underprivileged rural communities across Latin America occurs due to the domiciliary infestation with hematophagous triatomine bugs, which carry *T. cruzi* in the guts and transmit when the bite site or intact mucous membranes are contaminated with infected bug feces [6, 7]. In non-endemic regions, transmission can happen through organ transplantation or blood transfusion, and from an infected mother, congenital transmission can occur [7]. During acute infection, the damage to organs and tissues results from both the parasite *T. cruzi* itself and the elicitation of the host’s acute immunoinflammatory response by the presence of the parasite [8]. The course of the disease in the chronic phase is influenced by the balance between immune-mediated containment of the parasite and damage inflicting inflammation of the host tissues [1].

During the acute phase, the majority of the patients remain asymptomatic or experience mild, non-specific symptoms like fever, which makes them avoid clinical attention [6]. The chronic phase of Chagas disease is characterized by a broad spectrum of clinical presentations, ranging from the absence of any observable signs and symptoms of the disease (indeterminate form) to severe illness and premature mortality [1]. A wide range of clinical manifestations like arrhythmias, heart blocks, heart failure, sudden death, thromboembolism, and stroke occur during chronic Chagas cardiomyopathy [7, 9]. Direct observation of the parasite in the blood and/or molecular diagnosis are the initial diagnostic techniques in acute Chagas disease due to high levels of parasitemia [2]. At least two serological tests employing different methods with complementary sensitivity and specificity for detecting antibodies to *T. cruzi* are required for the confirmation of infection in the chronic phase [7, 9].

Currently, only two anti-trypanosomal drugs, benznidazole and nifurtimox, are proven to be effective against Chagas disease [10]. In chronic Chagas disease and Chagas cardiomyopathy, the role of anti-trypanosomal treatment remains controversial [10]. Despite its social, clinical, and epidemiological significance, the pathophysiology of chronic Chagas cardiomyopathy remains poorly understood, and the knowledge acquired from other cardiomyopathies still drives the therapeutic approach to chronic Chagas cardiomyopathy [11]. This article aims to explore and summarize the current state of knowledge concerning the pathophysiological mechanisms involved in the development and progression of chronic Chagas cardiomyopathy and shed light on the challenges and recent advances in its diagnostic techniques and treatment modalities.

## Review

Pathogenesis of Chagas cardiomyopathy

**Figure 1:**
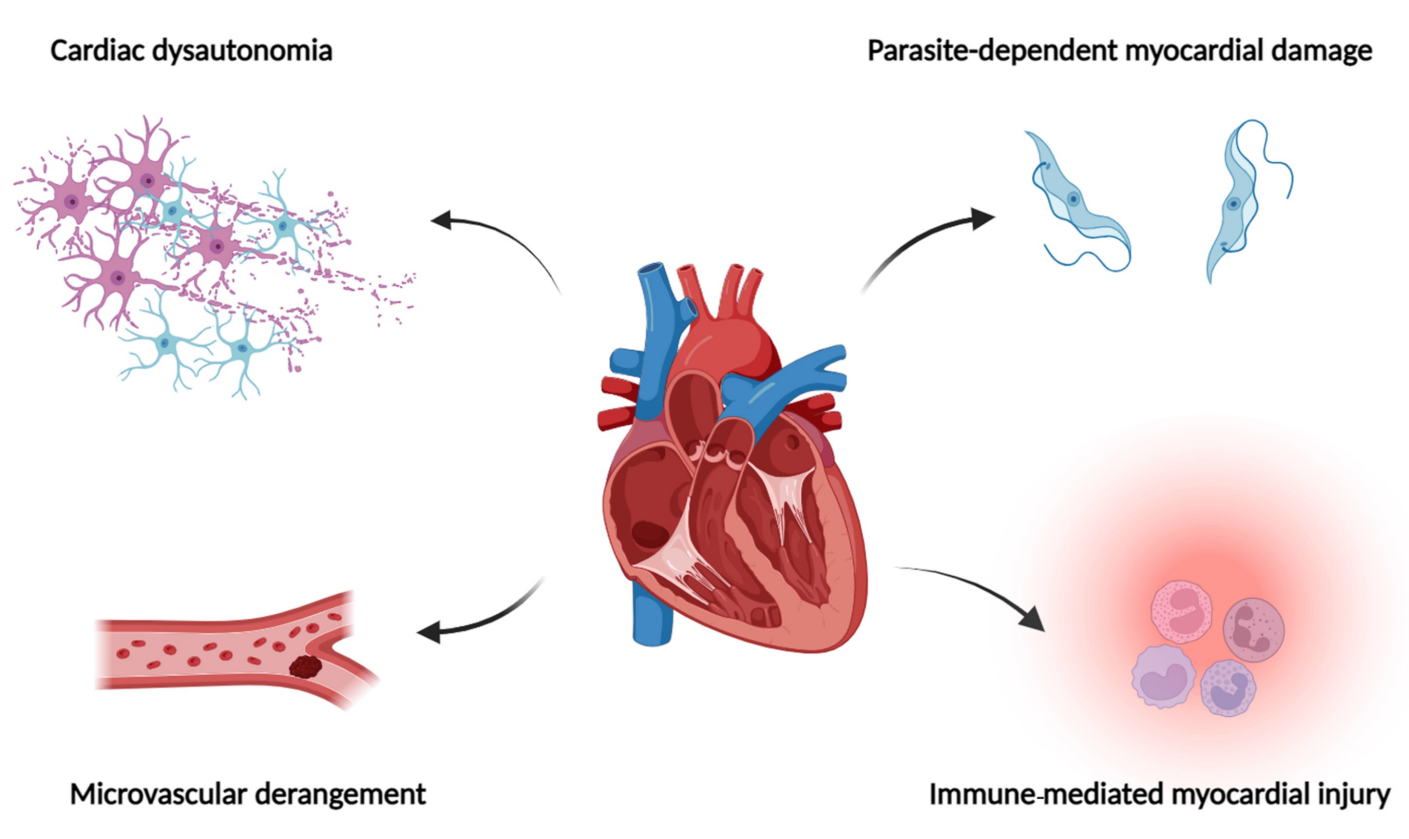
Pathogenesis of Chagas cardiomyopathy Image credits: Keerthana Veluswami Created with BioRender (BioRender, Toronto, Canada)

As mentioned earlier, only a few patients affected with Chagas disease develop chronic Chagas cardiomyopathy several years after the initial infection [12]. The susceptibility and progression to cardiomyopathy involve the interplay among four main pathogenetic mechanisms: cardiac dysautonomia, microvascular derangements, parasite-dependent myocardial damage, and immune-mediated myocardial injury (Figure 1) [12, 13]. Probably, the recurrent bouts of inflammation, which undergo periods of heightened exacerbation, are responsible for progressive neuronal damage, alterations in the microcirculation, deformations in the heart matrix, and subsequent organ failure [14].

Several factors responsible for the autonomic dysfunction in Chagas cardiomyopathy include neuronal depopulation, circulating antibodies that bind to beta-adrenergic and muscarinic receptors and down-regulate them, and periganglionic inflammation [13, 15, 16]. Cardiac dysautonomia is involved in different aspects of the development of cardiomyopathy [13]. Malignant arrhythmias and sudden death are due to early parasympathetic denervation [17]. Baroldi et al. conducted a study on the quantitative evaluation of the morphological lesions in the hearts of 34 serum-positive Chagas disease patients who died suddenly without any clinical evidence of cardiac disease. The study was published in 1997. The study concluded that the sudden death may be explained by the focal denervation, asynergy, and subsequent myotoxicity and malignant arrhythmias due to the compensatory adrenergic stimuli supported by the histological evidence of lymphocytic infiltrate and catecholamine-induced myocardial damage [17]. Several studies have demonstrated areas of dyssynergia in both the ventricles of a parasympathetically denervated chagasic heart. These dyssynergic areas lead to stressful adaptations in patients to intrinsically physiological conditions, which results in increased afterload and dilation of cardiac chambers [13]. Marin-Neto et al. conducted a study on 10 Chagas cardiomyopathy patients in 1986. Out of 10, five had parasympathetic denervation of the heart, and the other five had normal vagal regulation of heart rate. The study concluded that the pressor response to handgrip exercise in parasympathetically denervated patients is predominantly mediated not by an increase in cardiac output but by the rise in systemic vascular resistance [18]. It is also believed that dysautonomia can induce microcirculatory vasospasm [13]. Cardiac sympathetic denervation also occurs in addition to parasympathetic denervation [19]. Supporting the above statement, reports on chronic Chagas cardiomyopathy patients using 123I-metaiodobenzyl-guanidine scintigraphy showed segmental sympathetic denervation in the myocardium [20]. Machado et al. conducted a study on the histochemical evaluation of the nerve terminals in congestive heart failure (CHF) patients, which was published in 2000. The study was done on heart tissue specimens from 19 patients who underwent heart transplantation or partial ventriculectomy due to CHF, out of which 11 of them had Chagas disease. The study concluded that both sympathetic and parasympathetic nerve terminals were reduced in the hearts of all patients but with more severe parasympathetic denervation in Chagas cardiomyopathy [15]. Recent studies have proposed a new hypothesis that cardiac autonomic denervation accentuates the inflammatory process in Chagas cardiomyopathy, further destroying the autonomic neurons and establishing a positive feedback loop (Figure 1) [19].

Alterations in the microvasculature result in the development of myocardial ischemia and eventually lead to myocardial fibrosis [7, 13]. The microvascular derangements in Chagas disease occur due to endothelial dysfunction, and it is associated with excessive platelet activation, microthrombi, and changes in vasomotor control, resulting in thrombus formation in cardiac, pulmonary, or cerebral vessels [7, 13]. Platelet aggregation and microvascular spasm in Chagas cardiomyopathy at the molecular level are mediated by pro-inflammatory factors like thromboxane A2 and endothelin-1 [21]. Sialic acid, the protective component found on the endothelial surface, is removed by neuraminidase produced by the parasite *T. cruzi*, further contributing to the aggregation of platelets and microvascular thrombosis [22]. Sympathetic overstimulation also causes microcirculatory changes, resulting in microinfarctions, which have been postulated to contribute to the development of ventricular aneurysms [13, 23]. This theory of microvascular derangement requires further support in the form of a prospective cohort study that shows the effectiveness of long-term vasodilators or antiplatelet therapy on the clinical progression of chagasic patients presenting with angina-like symptoms [13]. Also, no prospective cohort studies have been conducted to establish a correlation between the presence and severity of myocardial perfusion defects and the temporal progression of segmental left ventricular wall motion abnormalities in patients with Chagas disease (Figure 1) [13].

*Trypanosoma cruzi* enters the cardiomyocyte by restricting the inhibitory effect of surface glycoconjugates like galectin-1 by altering it [10]. During the acute phase, the parasitic load and tissue tropism, based on the genetic characteristics of the host and the parasite, determine the parasite-dependent myocardial damage and clinical manifestations of the disease [10]. The mechanism of parasite-dependent myocardial damage during the acute phase includes mechanical rupture of infected cells, waste product release, luring of inflammatory cells, or acid-active hemolysin (TC-TOX) and LYT1, toxic products produced by *T. cruzi* [12, 24, 25]. During the chronic phase, the persistence of the parasite antigens detected by immunohistochemistry and polymerase chain reaction (PCR) drives the pathogenesis of myocardial damage [26, 27]. Belotti et al. conducted a study on 16 chronic Chagas cardiomyopathy patients and published it in 1998. The results showed the presence of *T. cruzi* antigens in 11 (69%) patients. Of 14 regions with histopathological evidence of moderate or severe myocarditis, 10 (71%) had *T. cruzi* antigens. In contrast, only three of 18 regions with mild or absent myocarditis showed the presence of *T. cruzi* antigens. The study concluded the persistence of *T. cruzi* antigens in chronic Chagas cardiomyopathy patients and its association with the degree of myocardial inflammation [27]. Still, the exact mechanism through which the myocardial tissue damage, mediated by the parasite during the chronic phase remains unclear (Figure 1) [13].

Myocardial injury and elevated heart failure risk occur due to both adaptive and innate immune responses induced by prolonged and sustained inflammation, oxidative stress injury, myofibril disruption, necrosis of myocytes, microvascular derangements, cardiac dysautonomia, hypertrophy of the heart, and fibrosis [24]. A delayed type IV hypersensitivity reaction characterized by diffuse mononuclear cell infiltrate is the hallmark of chronic Chagas cardiomyopathy [10]. Autoimmunity after *T. cruzi* infection is the reason for myocardial injury, and several mechanisms that induce autoimmunity have been proposed [28]. They are as follows: (A) molecular mimicry: the parasite antigens and the host antigens share epitopes of similar structures, which generates cross-reactivity due to their recognition by T and B cells; (B) polyclonal activation: production of autoantibodies; and (C) bystander activation: sensitization of the antigens released due to tissue damage in the inflammatory environment [13]. Cardiac damage has been hypothesized due to the breach of immunologic tolerance [13]. Cross-reacting antibodies have been detected due to the structural similarity between *T. cruzi* ribosomal P proteins and the human beta (β)1- adrenergic receptors [29]. The sera of patients with Chagas cardiomyopathy frequently demonstrated cross-reacting antibodies between the heavy chain of cardiac myosin and *T. cruzi* protein B13 compared to patients in the indeterminate phase [30]. Polyclonal lymphocyte activation can be triggered by at least one *T. cruzi *antigen, which acts as a B cell mitogen [31]. This polyclonal lymphocyte activation dampens the development of an intense, parasite-specific immune response, thereby reducing the efficiency of parasite clearance and increasing myocardial injury [32, 33]. Autoreactive T cells capable of initiating autoimmunity by overcoming self-tolerance are activated owing to the threshold lowering due to the surplus release of self-antigens in an environment packed with chemokines, inflammatory cytokines, lymphotoxin, and nitric oxide [25]. Cardiac inflammation can occur due to the highly immunogenic oxidized cardiac proteins that are formed as the result of *T. cruzi *infection, which leads to reduced cardiac contractility [24]. Treatment with the antiparasitic drug benznidazole reduced parasitemia, which in turn considerably reduced or eliminated the autoimmune response against myosin [34]. This shows that bystander activation can be reduced by reducing the parasite load, which in turn reduces the lysis of myocytes due to the parasite and the release of host antigens, thereby ameliorating the inflammatory environment induced by the infection [25]. The upsurge in the concentration of cytokines like interferon-γ and the recruitment and multiplication of *T. cruzi*-specific T cells to the myocardium, predominantly T helper (Th)1, is due to the persistence of the parasite [13, 24]. The predominance of Th1 cytokines like interferon-γ with a reduction in Th2 cytokines like interleukin (IL)-4 [35, 36] and high plasma levels of tumor necrosis factor-α characterize the chronic *T. cruzi* infection [37]. Gomes et al. conducted a study between 1995 and 2000 in Brazil, and the article was published in 2003. The study population consisted of 111 patients in the chronic phase of Chagas disease. People with the presence of the following conditions were excluded: alcoholism, diabetes mellitus, systemic arterial hypertension, renal insufficiency, thyroid dysfunction, hydro-electrolytic disorders, chronic obstructive pulmonary disease, history revealing coronary artery obstruction, rheumatic disease, and the individuals unable to undergo examinations. The results showed high interferon-γ levels in 83% of patients with the cardiac form of the disease and 59% of the patients in the indeterminate phase of Chagas disease. The study concluded that excess production of interferon-γ, which leads to heightened Th1 response, is associated with the progression of Chagas cardiomyopathy [37]. Regulatory T cells produce transforming growth factor (TGF)-β, a regulatory cytokine that causes the differentiation of naïve T cells into Th17 cells [38]. The reduced expression of this TGF-β due to genetic polymorphisms is linked to reduced susceptibility to Chagas cardiomyopathy [38]. Calzada et al. conducted a study on Peruvian and Colombian populations and published the article in 2009. The study included 347 seropositive and 279 seronegative individuals from both populations where *T. cruzi *is endemic. A significant difference in the distribution of the TGF-β1 gene's 10C and 10T alleles was noted in both the patients and healthy individuals in both populations. The study showed that seropositive cohorts in both populations had an increase in the frequency of high TGF-β1 producer genotype 10 C/C [38]. Autoimmunity has been established as one of the significant mechanisms in the pathogenesis of Chagas heart disease as a result of three decades of extensive research, yet the precise contribution of autoimmunity to disease progression is yet to be fully understood (Figure 1) [25].

The expression of antioxidant proteins is regulated by the transcription factor called nuclear factor (erythroid 2)-like 2 (NFE2L2) [24]. The studies have shown that the promotion of genes that cause fibrosis and the evolution of chronic Chagas cardiomyopathy is due to the inhibition of the NFE2L2/ antioxidant response element pathway by the mitochondrial reactive oxygen species (mtROS) [39]. Induction of thromboxane A, a prothrombotic lipid, increases parasitic load and inflicts damage to the cardiovasculature [40]. Activation of the kallikrein-kinin system increases myocardial parasitism and leads to a pro-inflammatory response brought about by vasodilation, increased mast cell expansion, an increase in vascular permeability, and cardiac edema, further promoting Chagas cardiomyopathy [41].

Clinical manifestations of Chagas cardiomyopathy

Clinical manifestations of Chagas disease can be categorized into two main phases: acute and chronic [10]. The acute phase manifestations occur after primary infection, from days eight to 10, where most of them, close to 90% to 95%, will remain asymptomatic [42]. Non-specific symptoms like fever, myalgia, arthralgia, headache, asthenia, adynamia, hepatomegaly, or splenomegaly occur in the other 5% of the patients [43]. Within eight to 12 weeks after transmission, the resolution of the acute phase occurs [10]. During the acute phase, cardiovascular manifestations like heart failure due to inflammation of the myocardium and pericardium and abnormalities of the conduction system are rare (~1%) and contribute to lower than 5% mortality [2, 42].

The patients progress into the chronic phase when they are not treated during the acute phase or when the parasitic infection is not successfully cleared by their immune system [10]. The patients in the chronic phase can either remain in the indeterminate stage (70%-90%) or develop the determinate stage (10%-30%), which is characterized by complications of cardiac, digestive, or cardio-digestive systems depending on their immunological response to the infection [44]. A positive serology of anti-*T. cruzi* antibodies, lack of any clinical manifestations of the disease, a normal ECG, and absence of any relevant findings during the imaging of the heart, esophagus, or colon define the indeterminate stage [7]. Since the patients remain asymptomatic during the indeterminate stage, it is challenging to identify this stage, and the disease often remains underdiagnosed [21].

**Figure 2:**
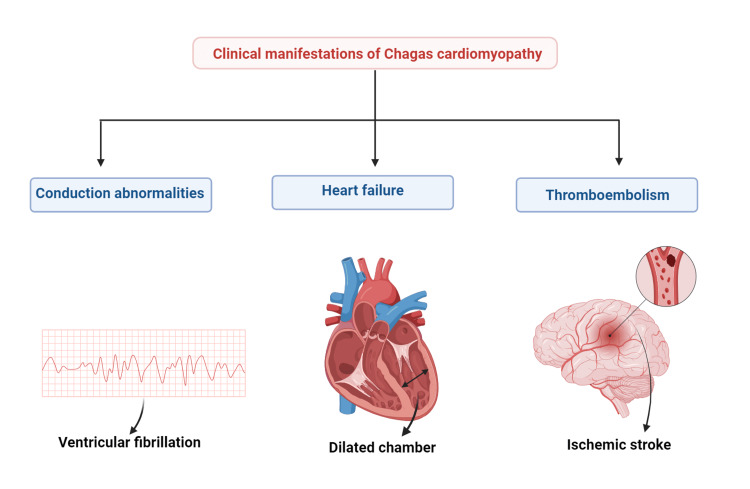
Clinical manifestations of Chagas cardiomyopathy Image credits: Keerthana Veluswami Created with BioRender (BioRender, Toronto, Canada)

Chagas cardiomyopathy is the most frequent type of manifestation during the determinant phase, and it includes abnormalities of the conduction system, heart failure, and thromboembolism (Figure 2) [42].

Conduction System Abnormalities

The cardiac conduction system, including the sinus node, atrioventricular node, and bundles of His, are selectively affected by fibrosis, and in these areas, macro-reentrant circuits are subsequently created, leading to conduction system abnormalities [7, 45]. The myocardial sympathetic defects predominantly affect the ventricular myocardium and contribute to arrhythmias [46]. Multiform premature ventricular contractions, right bundle branch block, or left anterior fascicular block are the earliest conduction abnormalities noted [47, 48]. More serious arrhythmias like sinus node dysfunction-induced severe bradycardia, atrial fibrillation, atrial flutter, atrioventricular block, premature ventricular contractions, and ventricular tachycardia, which can be either sustained or non-sustained, can occur due to the strong arrhythmogenic potential and progressive nature of the disease, causing permanent changes in the conduction system [47, 48]. Significant morbidity and mortality occur due to the role of ventricular tachycardia in sudden cardiac death (SCD) [10].

Heart Failure

Progressive dilated cardiomyopathy causes heart failure in Chagas disease. In the dilated heart, regional wall motion abnormalities are the first event to occur, followed by global left ventricular dysfunction. The left ventricle apex and the inferolateral wall are the commonly affected segments [49, 50]. Remodeling of the heart leads to Chagas cardiomyopathy [51]. Hiss et al. conducted a longitudinal observational study on 36 Chagas cardiomyopathy patients and published it in 2009. The study established that the increase in perfusion defects and myocardial fibrosis showed the progression of both segmental and global systolic dysfunction in the same regions [52]. Cardiac function is impaired during both systole and diastole [10]. Chronic inflammation of the myocardium first impairs the relaxation of the ventricles and their filling during diastole, and later, with the progression of the disease, systolic dysfunction occurs [53]. Exertion-related breathlessness and fatigue are the earliest symptoms of heart failure. Later, signs and symptoms of pulmonary and systemic congestion become prominent as the disease progresses. Further, paroxysmal nocturnal dyspnea, orthopnea, jugular venous distension, enlargement of the liver, edema of the lower limb, and generalized swelling of the whole body appear [54].

According to the Latin American Guidelines for Diagnosis and Treatment of Chagas disease, heart failure due to Chagas cardiomyopathy can be categorized into four stages: A, B (B1, B2), C, and D (Table 1) [55]. Stage A includes patients in indeterminate form who remain asymptomatic but carry the risk of developing Chagas cardiomyopathy and no evidence of structural heart disease (normal ECG and chest X-ray). Stage B comprises asymptomatic patients with either ECG or echocardiographic findings indicating structural heart disease. B1 patients have mild changes, but global ventricular function is preserved. B2 patients exhibit global ventricular dysfunction (reduced left ventricular ejection fraction). Stage C includes patients with heart failure symptoms currently or previously due to severe left ventricular dysfunction. Stage D includes patients who develop heart failure symptoms refractory to medical treatment, thus indicating the need for more specialized and advanced treatment [55].

**Table 1:** Stages of development of Chagas cardiomyopathy ECG: electrocardiogram; NYHA: New York Heart Association

Stage	Characteristic features	
Stage A	Patients in indeterminate form who remain asymptomatic but carry the risk of developing Chagas cardiomyopathy and no evidence of structural heart disease (normal ECG and chest X-ray).	
Stage B	Asymptomatic patients with either ECG or echocardiographic findings indicating structural heart disease.	B1: Patients have mild changes, but global ventricular function is preserved.
		B2: Patients exhibit global ventricular dysfunction (reduces left ventricular ejection fraction).
Stage C	Patients with heart failure symptoms currently or previously due to severe left ventricular dysfunction. (NYHA I, II, III, and IV)	
Stage D	Patients who develop heart failure symptoms refractory to medical treatment, thus indicating the need for more specialized and advanced treatment. (NYHA IV)	

Thromboembolism

During the chronic phase of Chagas disease, the patients are affected by systemic embolism, including stroke [54]. The causative factors include dilated cardiac chambers and left ventricle aneurysms, which favor the formation of intramural thrombi, ventricular dysfunction leading to blood flow stasis, and atrial conduction abnormalities [10]. In 8% of asymptomatic patients and 50% of patients with moderate to severe myocardial involvement, cardiac aneurysms involving the left ventricular apex are noted [7, 9]. Patients present with a motor or sensory deficit, aphasia, dysarthria, and vision changes [56]. Stroke in Latin American patients should raise suspicion of Chagas disease, and it should be included in their differential diagnosis because Chagas disease is regarded as the frequently unrecognized cause of stroke [9]. Thrombi formation in the venous system or right-sided cardiac chambers can lead to pulmonary embolism [10].

Diagnosis of Chagas cardiomyopathy

The World Health Organization and the Pan American Health Organization have formulated a criterion for the confirmation of Chagas disease, which includes two positive results obtained using two different serological methods [55]. Every patient should be evaluated initially with an ECG, and its abnormalities indicate cardiac involvement. According to presenting symptoms, patients can be subjected to further imaging modalities like echocardiography, chest X-rays, 24-hour Holter monitoring, exercise stress testing, electrophysiologic studies, nuclear medicine testing, cardiac magnetic resonance imaging (CMRI), or cardiac catheterization and coronary angiography [7].

Electrocardiogram

In patients with either a confirmed or suspected diagnosis of Chagas disease, ECG makes the most vital test in the preliminary evaluation. Specific typical ECG abnormalities increase clinical suspicion when present in people from the endemic region, and a serological test is ordered further to confirm it [7]. An ECG should be repeated at regular intervals despite an abnormal ECG in the beginning because the emergence of new ECG abnormalities marks the cardiomyopathy progression [57, 58]. The typical ECG abnormality found in Chagas disease is the combination of right bundle branch block and left anterior hemiblock, and it is less prevalent in other cardiomyopathies (Table 2) [59, 60]. Studies showing the prevalence of right bundle branch block associated with left anterior fascicular block in Chagas disease patients are summarized in Table 2.

**Table 2:** Studies showing the prevalence of right bundle branch block associated with left anterior fascicular block in Chagas disease patients ECG: electrocardiogram; *T. cruzi*: T*rypanosoma cruzi*; OR: odds ratio; CI: confidence interval

Reference	Study Design	Study Population	Results
Rodriguez et al. (2013) [60]	Cross-sectional study	455 heart disease patients, out of which 76 were seropositive for *T. cruzi*	The study found an association between seropositivity and the right bundle branch block (p = 0.004).
Marcolino et al. (2015) [61].	Retrospective observational study	264,324 participants underwent ECG during the study period, out of which 7,590 were Chagas disease patients	OR of 10.7 (95% CI – 10.1 to 11.4) for the right bundle branch block improved to OR of 12.1 (95% CI - 11.2 to 13.0) when associated with left anterior hemiblock.
Ribeiro et al. (2013) [62].	-	499 seropositive blood donors for *T. cruzi* infection and 483 seronegative donors as controls	A combination of right bundle branch block and left anterior fascicular block was more commonly found in seropositive donors (p<0.001)
Cardoso et al. (2016) [63].	Meta-analysis	17,238 patients, of whom positive serology for Chagas disease was found in 6,840 patients	In Chagas cardiomyopathy patients, there is an increased prevalence of the combination of right bundle branch block and left anterior fascicular block with the highest OR when compared to non-Chagas disease patients (OR 5.31; 95% CI 1.23-22.86; P = 0.03).
Rojas et al. (2018) [64].	Meta-analysis and systematic review	34,023 individuals, out of which 12,276 had Chagas disease	A combination of complete right bundle branch block and left anterior fascicular block (OR = 3.34; 95% CI = 1.76-6.35) was more prevalent in participants with Chagas disease than with non-Chagas disease participants.

Marcolino et al. conducted a retrospective observational study in Brazil between January 1 and December 31, 2011, and published it in 2015. The study comprised 264,324 patients who underwent ECG during the study period, of which 7,590 were Chagas disease patients. The results showed an odds ratio (OR) of 10.7 (95% confidence interval (CI) - 10.1 to 11.4) for the right bundle branch block, which improved to an OR of 12.1 (95% CI- 11.2 to 13.0) when associated with the left anterior hemiblock [61]. Ribeiro et al. conducted a study on 499 seropositive blood donors for *T. cruzi* infection and 483 seronegative donors as controls in Brazil and published it in 2013. The results showed that either as a separate entity or in association, the right bundle branch block and left anterior fascicular block were more commonly found in seropositive donors (p<0.0001) [62]. Cardoso et al. conducted a meta-analysis of 17,238 patients, of whom positive serology for Chagas disease was found in 6,840 patients and published in 2016. The study found that in Chagas cardiomyopathy patients, there is an increased prevalence of the combination of right bundle branch block along with left anterior fascicular block with the highest odds ratio (OR) when compared to non-Chagas disease patients (OR 5.31; 95% CI 1.23-22.86; P = 0.03) [63]. Rojas et al. conducted a meta-analysis and systematic review in 2018 on 34,023 individuals from 49 studies, of which 12,276 had Chagas disease. The article came into publication in 2018. The study showed that the combination of complete right bundle branch block along with left anterior fascicular block (OR = 3.34; 95% CI = 1.76-6.35) was more prevalent in participants with Chagas disease than non-Chagas disease participants [64].

The ECG features like duration of the maximum heart rate-corrected QT interval and QT interval dispersion, pathological Q waves, deviation of T wave axis, and presence of sustained ventricular tachycardia can serve as the predicting factors of mortality and severity [65, 66]. Ribeiro et al. studied 499 blood donors who were seropositive for *T. cruzi* and another 483 seronegative donors as controls in Brazil and published the study in 2013. The study reported that reduced left ventricular ejection fraction in Chagas disease can be predicted with moderate accuracy with ECG findings like QRS duration >120ms and a QT interval > 440ms. The study also found the common abnormalities associated with left ventricular ejection fraction in Chagas disease, like atrial fibrillation, ventricular extrasystole, right bundle branch block, old myocardial infarction, supraventricular premature beats, and isolated abnormalities of ST-T wave [62]. Holter monitoring is compulsory for Chagas disease patients who have premature ventricular contractions on ECG as they are prone to develop complex ventricular arrhythmias [7].

Echocardiography

The left ventricular systolic and diastolic dysfunction, involvement of the right ventricle, regional abnormalities in contractility, and the pathognomonic apical aneurysm in Chagas cardiomyopathy can be recognized by echocardiography, a frequently used non-invasive imaging modality [67]. The common echocardiographic finding in the acute phase of Chagas disease is the presence of pericardial effusion [21]. Even if the previous ECG was normal during the indeterminate phase, echocardiography may find segmental wall motion abnormalities in the left ventricle. As the disease progresses, cardiac chamber dilatation and global hypokinesia can be detected. Cardiomegaly can lead to secondary mitral and/or tricuspid regurgitation due to valve annuli dilatation [54]. When atrial fibrillation and other supraventricular tachyarrhythmias are present, transthoracic and transesophageal echocardiograms are used together to rule out thrombi in atria and ventricles [10]. Chagas cardiomyopathy typically involves the basal segments of the inferior and inferolateral wall and the cardiac apex, and changes in these regions cannot be associated with obstructive coronary artery disease [68].

Magnetic Resonance Imaging

A CMRI is superior to other imaging techniques like echocardiograms because it accurately measures both right and left ventricle ejection fractions, regional wall motion abnormality detection, tissue catheterization, aneurysms, and thrombi [10]. Patients with extensive myocardial fibrosis causing global or regional dysfunction of the ventricles and at increased risk of ventricular tachycardia can be picked up by CMRI using the delayed enhancement technique [69]. Rochitte et al. conducted a study on 51 patients at various stages of Chagas disease. Out of these, 15 were in the indeterminate phase, 26 had established clinical Chagas heart disease, and 10 had Chagas heart disease and ventricular tachycardia and published the study in 2005. The study results showed the presence of myocardial fibrosis using myocardial delayed enhancement (MDE) by MRI in 20% of indeterminate phase patients, 84.6% of Chagas heart disease patients, and 100% of patients with Chagas heart disease and ventricular tachycardia. So, the study concluded that myocardial fibrosis in Chagas heart disease can be detected even in the asymptomatic indeterminate phase using MDE by MRI, and MRI helps predict the severity of the disease and the impending risk of SCD [70].

Other Diagnostic Techniques

Coronary perfusion can be assessed by cardiopulmonary exercise testing and myocardial perfusion scintigraphy. In patients who presented with anginal pain and normal coronary angiography, scintigraphy showed segmental perfusion defects in 30% of them [55]. In symptomatic patients for whom no pathophysiological explanation can be given by non-invasive diagnostic techniques, electrophysiological studies can aid in assessing the function of the sinus node and atrioventricular conduction [55]. The feasibility of heart transplantation in patients who are eligible for the procedure can be determined by cardiac catheterization, which helps in measuring pulmonary vascular resistance [7]. Nuclear imaging techniques like single photon emission computed tomography and 18F-fluorodeoxyglucose positron emission tomography are the current areas of research as they possess the capability to detect and monitor subclinical damage to the myocardium in the earliest stages, thus aiding in treatment modalities targeting inflammation and microvascular ischemia, and this helps in the better categorization of Chagas heart disease patients based on their prognosis [71].

Biomarkers

In different clinical forms of Chagas disease, specific inflammatory and cardiac biomarkers are differentially expressed, which may be helpful in the staging of the disease [10]. Keating et al. conducted a cross-sectional retrospective case-control study on over 1000 individuals, out of which 449 were seropositive for *T. cruzi* and another 101 had clinical Chagas cardiomyopathy, to test 22 biomarkers and published it in 2015. The study found significant levels of inflammatory biomarkers IL-10 and IL-6 and cardiac dysfunction markers like troponin, N-terminal of the prohormone brain natriuretic peptide (NT-pro-BNP), myoglobin, creatine kinase-MB, and adiponectin in Chagas cardiomyopathy patients. Disease progression can be detected in the early stage using troponin and NT-pro-BNP as their levels increased in Chagas disease patients without heart disease. NT-pro-BNP strongly predicted the New York Heart Association (NYHA) functional class, ventricular arrhythmia, and dysfunction of the left ventricle [72].

Treatment of Chagas cardiomyopathy

Like any other chronic cardiac disease, Chagas cardiomyopathy is managed through an interdisciplinary approach that encompasses lifestyle modification and pharmacological treatment, which form the pillars of its treatment [73].

Antiparasitic Treatment

The antiparasitic treatment with the drugs benznidazole and nifurtimox is prescribed for all patients during the acute phase [54]. At present, the role of anti-trypanosomal drugs in the treatment of Chagas cardiomyopathy lacks supporting evidence [73]. Given this, a multicenter, international, controlled clinical trial named ‘Benznidazole Evaluation for Interrupting Trypanosomiasis’ was conducted between 2004 and 2011. This trial randomized 2,854 patients to be treated with either placebo or benznidazole. The benznidazole group showed a significant reduction in parasitemia but no difference in clinical outcomes such as SCD, pacemaker requirement, mortality, insertion of implantable defibrillator, heart transplant, heart failure, and stroke [73].

Neuro-Hormonal Blockade

Blocking neuro-hormonal pathways and preventing SCD are the primary treatment approaches [74]. Chagas heart failure treatment is derived from the knowledge acquired from the data on the treatment of heart failure due to non-Chagas disease. Drugs like angiotensin-converting enzyme inhibitors (ACE-I) or angiotensin II receptor blockers, β-blocker, and mineralocorticoid receptor antagonists should be given to patients with NYHA III or IV functional class or after assessing the left ventricular ejection fraction [74]. Due to their adrenergic response modulation, β-blockers play a crucial role in symptomatic heart failure patients with reduced left ventricular ejection fraction. Still, their use is limited as the Chagas cardiomyopathy patients have low baseline heart rates or due to amiodarone use. However, studies suggest that before starting any antiarrhythmic drug, β-blockers should be titrated to their maximum dose [75]. In patients suffering from heart failure caused by Chagas cardiomyopathy, it is recommended to use ACE-I and diuretics initially to compensate for symptoms of congestion, and later on, β-blockers can be added safely at targeted doses [75]. A double-blind, randomized control trial named ‘Prospective Comparison of Angiotensin Receptor Neprilysin Inhibitor With ACE-I to Determine the Impact on Global Mortality and Morbidity in Heart Failure’ was conducted in patients in whom left ventricular ejection fraction reduced heart failure was present, to compare sacubitril/valsartan against enalapril. The study randomized 113 Chagas cardiomyopathy patients, with 58 to the sacubitril/valsartan group and 55 to enalapril. Hospitalization due to heart failure and mortality due to cardiovascular causes were low with sacubitril/valsartan compared to enalapril. A strong recommendation was not established as the trial was underpowered [76].

Prevention of SCD

Chagas disease patients with sustained ventricular tachycardia, without considering left ventricular ejection fraction, and who have recovered from cardiac arrest are recommended with an implantable cardioverter-defibrillator (ICD) [77]. Prevention of SCD is achieved by the implantation of an ICD with amiodarone, which decreases the incidence of life-threatening arrhythmias [78]. Gali et al. conducted an observational study on 76 Chagas cardiomyopathy patients in 2014. There was a 72% reduction in all-cause mortality risk (P = 0.007) and a 95% reduction in SCD risk (P = 0.006) in patients who were treated with amiodarone plus ICD compared to amiodarone alone (Table 3) [78].

**Table 3:** Outcomes of Chagas heart disease patients treated with ICD CHD: Chagas heart disease; VA: ventricular arrhythmias; ICD: implantable cardioverter-defibrillator; SCD: sudden cardiac death

Reference	Type of study	Population	Conclusion
Gali et al. (2014) [78]	Retrospective observational study	ICD + amiodarone group: 76 CHD patients with life-threatening VA; Amiodarone group: 28 CHD patients with life-threatening VA	ICD plus amiodarone treatment was effective in reducing all-cause mortality and SCD in CHD patients with life-threatening VA compared to amiodarone-only treatment.
Pavão et al. (2018) [79]	Retrospective observational study	111 CHD patients with ICD	ICDs were successful in aborting life-threatening arrhythmias in CHD patients. 50 (45%) of the patients died with an annual mortality rate of 8.4%, either due to refractory heart failure or due to non-cardiac causes.
Pereira et al. (2014) [80]	-	65 CHD patients with ICD	ICD effectively prevented SCD in chronic CHD patients. A total of 13 (20%) patients died (annual mortality rate was 6.1%), and no sudden death was reported.
Martinelli et al. (2012) [81]	Retrospective cohort study	116 CHD patients with ICD	In CHD patients, ICD was efficient in the secondary prevention of SCD after long-term follow-up with a 7.1% all-cause mortality rate.

Ventricular tachycardia that is recurrent or refractory to antiarrhythmic treatment can be managed using surgical or catheter-based ablation [74]. Implantation of a pacemaker can be considered for symptomatic sick sinus syndrome or in advanced atrioventricular blocks [10].

Advanced Treatment Options

Patients with end-stage Chagas cardiomyopathy can be suggested with a left ventricular assist device as a transitional therapy to heart transplantation or as the final treatment [10].

For Chagas cardiomyopathy patients with heart failure refractory to medical management, a heart transplant is an alternative treatment option [54]. The survival rate at the end of one year is 71%, and at the end of 10 years post transplant is 46% [54]. Immunosuppression following heart transplantation is associated with *T. cruzi *reactivation [10]. Only very low mortality is associated with *T. cruzi* reactivation and can be treated easily. Compared to non-Chagas patients who received heart transplants, Chagasic patients showed a higher probability of survival [82]. To prevent clinically significant Chagas disease, monitoring for *T. cruzi* reactivation and initiating treatment is necessary [83]. Monitoring is done by the detection of *T. cruzi* DNA in blood using PCR and reviewing peripheral blood for the presence of the parasite in the following frequencies: every week for the first two months posttransplant, every two weeks from the third to the sixth month, and after that every month (Table 4) [84, 85].

**Table 4:** Monitoring for T. cruzi reactivation post heart transplant DNA: deoxyribonucleic acid; PCR: polymerase chain reaction; *T. cruzi*: *Trypanosoma cruzi*

Monitoring method 1: Detection of *T. cruzi *DNA in blood using PCR	
Monitoring method 2: Reviewing peripheral blood for the presence of the parasite	
Months after transplant	Periodicity of monitoring
Initial two months	Every week
Third to sixth month	Every two weeks
After six months	Every month

Rossi et al. conducted a case series in Brazil over 18 years from 1996 through 2014. The study included 53 Chagas disease patients who underwent heart transplants. Eighteen patients received prophylactic benznidazole therapy before transplant, out of which only two of them (11.1%) developed reactivation of Chagas disease. Meanwhile, in the group that did not receive prophylaxis, 45.7% of them had Chagas disease reactivation. This study shows that the reactivation of *T. cruzi* infection can be reduced by administering prophylactic therapy before heart transplantation (OR = 0.12) [86]. Randomized control trials are necessary to support and formulate a more adequate regimen [10].

Future Scope for Research

The possibility of reducing the oxidative stress created during Chagas disease using drugs that stimulate the NFE2L2 pathway is being evaluated as adjuvant therapy [24]. Normal cardiac histology and myocardial contractility can be maintained by spin-trapping antioxidant α-phenyl-N-tert-butyl nitrone by preventing the oxidation of proteins and the subsequent immunity to them [87]. Enhancement of regulatory T cell functionality using adoptive transfer of regulatory T cells, intravenous administration of immunoglobulin, low-dose IL-2 antibody complex employment, sphingosine 1-phosphate receptor 1 agonist administration, and supplementation of vitamin D [88]. However, the effectiveness of these therapies remains untested [24].

Limitations

This article solely reviews the pathological changes in the heart and clinical manifestations that arise due to the cardiac abnormalities due to *T. cruzi *infection while ignoring other co-existing factors that bring about the same changes like genetic predisposition, comorbidities, and environmental factors. Chagas disease also affects the esophagus and the colon, which this article has not discussed. Being endemic in the resource-limited rural areas of Latin America and high prevalence among the migrants in non-endemic regions made documentation difficult, and this has led to the data on the Chagas disease and its cardiovascular consequences being inaccurate. Certain studies reviewed in this article were conducted only on the sample population from endemic regions in Latin America; thus, the results from those studies cannot be generalized. This article did not mention all the trials currently being carried out to find an effective management plan.

## Conclusions

Due to its broad spectrum of varied presentations, Chagas cardiomyopathy implicated significant challenges in identifying the cases and delayed further research during the initial years. Cardiac denervation, microinfarctions due to perfusion defects, and direct myocardial injury inflicted by both parasite-dependent immunity and autoimmunity are the primary mechanisms involved in the pathogenesis of the disease. Recent studies have found the involvement of NFE2L2 pathway inhibition by the mtROS in the evolution of Chagas cardiomyopathy, and enhancing the same pathway is currently being studied as one of the potential therapeutic options. The basic diagnostic modality ECG helps in both raising clinical suspicion as well as in determining the prognosis of the disease. Biomarkers and nuclear imaging techniques will also prove to be an efficient tool in the early diagnosis and facilitate new treatment modalities for Chagas cardiomyopathy. The existing anti-trypanosomal drugs, benznidazole and nifurtimox, are of suboptimal efficacy. Thus, improved antimicrobial drugs should be explored. The complexity of treating Chagas cardiomyopathy in its advanced stages emphasizes the importance of large-scale clinical trials regarding this topic. This review article will serve as a collective tool for the medical fraternity to identify current gaps in knowledge regarding this topic and help create awareness among physicians, especially in non-endemic countries, to screen patients from endemic regions and patients from non-endemic areas with typical ECG findings. Finally, there is an absolute need to conduct more research to standardize the diagnostic criteria and frame an effective etiology-specific management plan.

## References

[1] Rassi A Jr, Rassi A, Marin-Neto JA (2010). Chagas disease. Lancet.

[2] Echeverría LE, Marcus R, Novick G (2020). WHF IASC roadmap on Chagas disease. Glob Heart.

[3] (2024). Chagas disease (also known as American trypanosomiasis). https://www.who.int/news-room/fact-sheets/detail/chagas-disease-(american-trypanosomiasis).

[4] Saraiva RM, Mediano MF, Mendes FS (2021). Chagas heart disease: an overview of diagnosis, manifestations, treatment, and care. World J Cardiol.

[5] Schmunis GA (2007). Epidemiology of Chagas disease in non-endemic countries: the role of international migration. Mem Inst Oswaldo Cruz.

[6] Bern C, Kjos S, Yabsley MJ, Montgomery SP (2011). Trypanosoma cruzi and Chagas' Disease in the United States. Clin Microbiol Rev.

[7] Nunes MC, Beaton A, Acquatella H (2018). Chagas cardiomyopathy: an update of current clinical knowledge and management: a scientific statement from the American Heart Association. Circulation.

[8] Andrade ZA (1999). Immunopathology of Chagas disease. Mem Inst Oswaldo Cruz.

[9] Nunes MC, Dones W, Morillo CA, Encina JJ, Ribeiro AL (2013). Chagas disease: an overview of clinical and epidemiological aspects. J Am Coll Cardiol.

[10] Pino-Marín A, Medina-Rincón GJ, Gallo-Bernal S (2021). Chagas cardiomyopathy: From Romaña sign to heart failure and sudden cardiac death. Pathogens.

[11] Botoni FA, Ribeiro AL, Marinho CC, Lima MM, Nunes Mdo C, Rocha MO (2013). Treatment of Chagas cardiomyopathy. Biomed Res Int.

[12] Velasco A, Morillo CA (2020). Chagas heart disease: a contemporary review. J Nucl Cardiol.

[13] Marin-Neto JA, Cunha-Neto E, Maciel BC, Simões MV (2007). Pathogenesis of chronic Chagas heart disease. Circulation.

[14] Higuchi Mde L, Benvenuti LA, Martins Reis M, Metzger M (2003). Pathophysiology of the heart in Chagas' disease: current status and new developments. Cardiovasc Res.

[15] Machado CR, Camargos ER, Guerra LB, Moreira MC (2000). Cardiac autonomic denervation in congestive heart failure: comparison of Chagas' heart disease with other dilated cardiomyopathy. Hum Pathol.

[16] Borda ES, Sterin-Borda L (1996). Antiadrenergic and muscarinic receptor antibodies in Chagas' cardiomyopathy. Int J Cardiol.

[17] Baroldi G, Oliveira SJ, Silver MD (1997). Sudden and unexpected death in clinically 'silent' Chagas' disease. A hypothesis. Int J Cardiol.

[18] Marin-Neto JA, Maciel BC, Gallo Júnior L, Junqueira Júnior LF, Amorim DS (1986). Effect of parasympathetic impairment on the haemodynamic response to handgrip in Chagas's heart disease. Br Heart J.

[19] Ribeiro Machado MP, Dias da Silva VJ (2012). Autonomic neuroimmunomodulation in chagasic cardiomyopathy. Exp Physiol.

[20] Simões MV, Pintya AO, Bromberg-Marin G (2000). Relation of regional sympathetic denervation and myocardial perfusion disturbance to wall motion impairment in Chagas' cardiomyopathy. Am J Cardiol.

[21] Montalvo-Ocotoxtle IG, Rojas-Velasco G, Rodríguez-Morales O (2022). Chagas heart disease: beyond a single complication, from asymptomatic disease to heart failure. J Clin Med.

[22] Libby P, Alroy J, Pereira ME (1986). A neuraminidase from Trypanosoma cruzi removes sialic acid from the surface of mammalian myocardial and endothelial cells. J Clin Invest.

[23] Marin-Neto JA, Simões MV, Rassi Junior A (2013). Pathogenesis of chronic Chagas cardiomyopathy: the role of coronary microvascular derangements. Rev Soc Bras Med Trop.

[24] Bonney KM, Luthringer DJ, Kim SA, Garg NJ, Engman DM (2019). Pathology and pathogenesis of Chagas heart disease. Annu Rev Pathol.

[25] Bonney KM, Engman DM (2008). Chagas heart disease pathogenesis: one mechanism or many?. Curr Mol Med.

[26] Higuchi Mde L, De Brito T, Martins Reis M, Barbosa A, Bellotti G, Pereira-Barreto AC, Pileggi F (1993). Correlation between Trypanosoma cruzi parasitism and myocardial inflammatory infiltrate in human chronic chagasic myocarditis: Light microscopy and immunohistochemical findings. Cardiovasc Pathol.

[27] Bellotti G, Bocchi EA, de Moraes AV (1996). In vivo detection of Trypanosoma cruzi antigens in hearts of patients with chronic Chagas' heart disease. Am Heart J.

[28] Cunha-Neto E, Bilate AM, Hyland KV, Fonseca SG, Kalil J, Engman DM (2006). Induction of cardiac autoimmunity in Chagas heart disease: a case for molecular mimicry. Autoimmunity.

[29] Ferrari I, Levin MJ, Wallukat G (1995). Molecular mimicry between the immunodominant ribosomal protein P0 of Trypanosoma cruzi and a functional epitope on the human beta 1-adrenergic receptor. J Exp Med.

[30] Cunha-Neto E, Duranti M, Gruber A (1995). Autoimmunity in Chagas disease cardiopathy: biological relevance of a cardiac myosin-specific epitope crossreactive to an immunodominant Trypanosoma cruzi antigen. Proc Natl Acad Sci U S A.

[31] Gao W, Wortis HH, Pereira MA (2002). The Trypanosoma cruzi trans-sialidase is a T cell-independent B cell mitogen and an inducer of non-specific Ig secretion. Int Immunol.

[32] Reina-San-Martín B, Degrave W, Rougeot C (2000). A B-cell mitogen from a pathogenic trypanosome is a eukaryotic proline racemase. Nat Med.

[33] Bryan MA, Norris KA (2010). Genetic immunization converts the trypanosoma cruzi B-Cell mitogen proline racemase to an effective immunogen. Infect Immun.

[34] Hyland KV, Leon JS, Daniels MD (2007). Modulation of autoimmunity by treatment of an infectious disease. Infect Immun.

[35] Ribeirão M, Pereira-Chioccola VL, Rénia L, Augusto Fragata Filho A, Schenkman S, Rodrigues MM (2000). Chagasic patients develop a type 1 immune response to Trypanosoma cruzi trans-sialidase. Parasite Immunol.

[36] Abel LC, Rizzo LV, Ianni B (2001). Chronic Chagas' disease cardiomyopathy patients display an increased IFN-gamma response to Trypanosoma cruzi infection. J Autoimmun.

[37] Gomes JA, Bahia-Oliveira LM, Rocha MO, Martins-Filho OA, Gazzinelli G, Correa-Oliveira R (2003). Evidence that development of severe cardiomyopathy in human Chagas' disease is due to a Th1-specific immune response. Infect Immun.

[38] Calzada JE, Beraún Y, González CI, Martín J (2009). Transforming growth factor beta 1 (TGFbeta1) gene polymorphisms and Chagas disease susceptibility in Peruvian and Colombian patients. Cytokine.

[39] Wen JJ, Porter C, Garg NJ (2017). Inhibition of NFE2L2-antioxidant response element pathway by mitochondrial reactive oxygen species contributes to development of cardiomyopathy and left ventricular dysfunction in Chagas disease. Antioxid Redox Signal.

[40] Tanowitz HB, Mukhopadhyay A, Ashton AW, Lisanti MP, Machado FS, Weiss LM, Mukherjee S (2011). Microarray analysis of the mammalian thromboxane receptor-Trypanosoma cruzi interaction. Cell Cycle.

[41] Nascimento CR, Andrade D, Carvalho-Pinto CE (2017). Mast cell coupling to the kallikrein-kinin system fuels intracardiac parasitism and worsens heart pathology in experimental Chagas disease. Front Immunol.

[42] Rassi A Jr, Rassi A, Marcondes de Rezende J (2012). American trypanosomiasis (Chagas disease). Infect Dis Clin North Am.

[43] Álvarez-Hernández DA, Franyuti-Kelly GA, Díaz-López-Silva R, González-Chávez AM, González-Hermosillo-Cornejo D, Vázquez-López R (2018). Chagas disease: current perspectives on a forgotten disease. Rev Med Hosp Gen (Mex).

[44] Bern C (2015). Chagas' disease. N Engl J Med.

[45] Rocha AL, Lombardi F, da Costa Rocha MO, Barros MV, Val Barros Vda C, Reis AM, Ribeiro AL (2006). Chronotropic incompetence and abnormal autonomic modulation in ambulatory Chagas disease patients. Ann Noninvasive Electrocardiol.

[46] Miranda CH, Figueiredo AB, Maciel BC, Marin-Neto JA, Simões MV (2011). Sustained ventricular tachycardia is associated with regional myocardial sympathetic denervation assessed with 123I-metaiodobenzylguanidine in chronic Chagas cardiomyopathy. J Nucl Med.

[47] Rassi A Jr, Dias JC, Marin-Neto JA, Rassi A (2009). Challenges and opportunities for primary, secondary, and tertiary prevention of Chagas' disease. Heart.

[48] Rassi A Jr, Rassi A, Little WC (2000). Chagas' heart disease. Clin Cardiol.

[49] Acquatella H, Schiller NB, Puigbó JJ (1980). M-mode and two-dimensional echocardiography in chronic Chages' heart disease. A clinical and pathologic study. Circulation.

[50] Pazin-Filho A, Romano MM, Almeida-Filho OC (2006). Minor segmental wall motion abnormalities detected in patients with Chagas' disease have adverse prognostic implications. Braz J Med Biol Res.

[51] Tanowitz HB, Machado FS, Spray DC (2015). Developments in the management of Chagas cardiomyopathy. Expert Rev Cardiovasc Ther.

[52] Hiss FC, Lascala TF, Maciel BC, Marin-Neto JA, Simões MV (2009). Changes in myocardial perfusion correlate with deterioration of left ventricular systolic function in chronic Chagas' cardiomyopathy. JACC Cardiovasc Imaging.

[53] Pinto Ade S, Oliveira BM, Botoni FA, Ribeiro AL, Rocha MO (2007). Myocardial dysfunction in chagasic patients with no apparent heart disease. Arq Bras Cardiol.

[54] Santos É, Menezes Falcão L (2020). Chagas cardiomyopathy and heart failure: from epidemiology to treatment. Rev Port Cardiol (Engl Ed).

[55] Andrade JP, Marin Neto JA, Paola AA (2011). I Latin American Guidelines for the diagnosis and treatment of Chagas' heart disease: executive summary (Article in Portugese). Arq Bras Cardiol.

[56] Carod-Artal FJ, Vargas AP, Horan TA, Nunes LG (2005). Chagasic cardiomyopathy is independently associated with ischemic stroke in Chagas disease. Stroke.

[57] Maguire JH, Hoff R, Sherlock I (1987). Cardiac morbidity and mortality due to Chagas' disease: prospective electrocardiographic study of a Brazilian community. Circulation.

[58] Nascimento BR, Araújo CG, Rocha MO, Domingues JD, Rodrigues AB, Barros MV, Ribeiro AL (2012). The prognostic significance of electrocardiographic changes in Chagas disease. J Electrocardiol.

[59] Bestetti RB, Muccillo G (1997). Clinical course of Chagas' heart disease: a comparison with dilated cardiomyopathy. Int J Cardiol.

[60] Rodriguez MV, Hernandez WY, Garcia AN, Colato CM, Cardoza PG, Cardozo LM (2013). ELISA seroprevalence of Trypanosoma cruzi in a cohort of heart disease patients. J Infect Dev Ctries.

[61] Marcolino MS, Palhares DM, Ferreira LR, Ribeiro AL (2015). Electrocardiogram and Chagas disease: a large population database of primary care patients. Glob Heart.

[62] Ribeiro AL, Sabino EC, Marcolino MS (2013). Electrocardiographic abnormalities in Trypanosoma cruzi seropositive and seronegative former blood donors. PLoS Negl Trop Dis.

[63] Cardoso R, Garcia D, Fernandes G (2016). The prevalence of atrial fibrillation and conduction abnormalities in Chagas disease: a meta-analysis. J Cardiovasc Electrophysiol.

[64] Rojas LZ, Glisic M, Pletsch-Borba L (2018). Electrocardiographic abnormalities in Chagas disease in the general population: a systematic review and meta-analysis. PLoS Negl Trop Dis.

[65] Salles G, Xavier S, Sousa A, Hasslocher-Moreno A, Cardoso C (2003). Prognostic value of QT interval parameters for mortality risk stratification in Chagas' disease: results of a long-term follow-up study. Circulation.

[66] Salles GF, Xavier SS, Sousa AS, Hasslocher-Moreno A, Cardoso CR (2004). T-wave axis deviation as an independent predictor of mortality in chronic Chagas' disease. Am J Cardiol.

[67] Biolo A, Ribeiro AL, Clausell N (2010). Chagas cardiomyopathy-where do we stand after a hundred years?. Prog Cardiovasc Dis.

[68] Acquatella H (2007). Echocardiography in Chagas heart disease. Circulation.

[69] Mello RP, Szarf G, Schvartzman PR (2012). Delayed enhancement cardiac magnetic resonance imaging can identify the risk for ventricular tachycardia in chronic Chagas' heart disease. Arq Bras Cardiol.

[70] Rochitte CE, Oliveira PF, Andrade JM (2005). Myocardial delayed enhancement by magnetic resonance imaging in patients with Chagas' disease: a marker of disease severity. J Am Coll Cardiol.

[71] Simões MV, Tanaka DM, Marin-Neto JA (2020). Nuclear medicine methods for assessment of chronic Chagas heart disease. Int J Cardiovasc Sci.

[72] Keating SM, Deng X, Fernandes F (2015). Inflammatory and cardiac biomarkers are differentially expressed in clinical stages of Chagas disease. Int J Cardiol.

[73] Medina-Rincón GJ, Gallo-Bernal S, Jiménez PA (2021). Molecular and clinical aspects of chronic manifestations in chagas disease: a state-of-the-art review. Pathogens.

[74] Bestetti RB, Theodoropoulos TA, Cardinalli-Neto A, Cury PM (2008). Treatment of chronic systolic heart failure secondary to Chagas heart disease in the current era of heart failure therapy. Am Heart J.

[75] Botoni FA, Poole-Wilson PA, Ribeiro AL (2007). A randomized trial of carvedilol after renin-angiotensin system inhibition in chronic Chagas cardiomyopathy. Am Heart J.

[76] Ramires FJ, Martinez F, Gómez EA (2018). Post hoc analyses of SHIFT and PARADIGM-HF highlight the importance of chronic Chagas' cardiomyopathy comment on: "Safety profile and efficacy of ivabradine in heart failure due to Chagas heart disease: a post hoc analysis of the SHIFT trial" by Bocchi et al. ESC Heart Fail.

[77] Meymandi S, Hernandez S, Park S, Sanchez DR, Forsyth C (2018). Treatment of Chagas disease in the United States. Curr Treat Options Infect Dis.

[78] Gali WL, Sarabanda AV, Baggio JM, Ferreira LG, Gomes GG, Marin-Neto JA, Junqueira LF (2014). Implantable cardioverter-defibrillators for treatment of sustained ventricular arrhythmias in patients with Chagas' heart disease: comparison with a control group treated with amiodarone alone. Europace.

[79] Pavão ML, Arfelli E, Scorzoni-Filho A (2018). Long-term follow-up of Chagas heart disease patients receiving an implantable cardioverter-defibrillator for secondary prevention. Pacing Clin Electrophysiol.

[80] Pereira FT, Rocha EA, Monteiro Mde P, Neto AC, Daher Ede F, Sobrinho CR, Pires Neto Rda J (2014). Long-term follow-up of patients with chronic chagas disease and implantable cardioverter-defibrillator. Pacing Clin Electrophysiol.

[81] Martinelli M, de Siqueira SF, Sternick EB, Rassi A Jr, Costa R, Ramires JA, Kalil Filho R (2012). Long-term follow-up of implantable cardioverter-defibrillator for secondary prevention in Chagas' heart disease. Am J Cardiol.

[82] Bestetti RB, Theodoropoulos TA (2009). A systematic review of studies on heart transplantation for patients with end-stage Chagas' heart disease. J Card Fail.

[83] Benatti RD, Oliveira GH, Bacal F (2017). Heart transplantation for Chagas cardiomyopathy. J Heart Lung Transplant.

[84] Chin-Hong PV, Schwartz BS, Bern C (2011). Screening and treatment of Chagas disease in organ transplant recipients in the United States: recommendations from the Chagas in transplant working group. Am J Transplant.

[85] Schwartz BS, Mawhorter SD (2013). Parasitic infections in solid organ transplantation. Am J Transplant.

[86] Rossi Neto JM, Finger MA, Dos Santos CC (2020). Benznidazole as prophylaxis for Chagas disease infection reactivation in heart transplant patients: a case series in Brazil. Trop Med Infect Dis.

[87] Dhiman M, Zago MP, Nunez S (2012). Cardiac-oxidized antigens are targets of immune recognition by antibodies and potential molecular determinants in chagas disease pathogenesis. PLoS One.

[88] Altara R, Mallat Z, Booz GW, Zouein FA (2016). The CXCL10/CXCR3 axis and cardiac inflammation: Implications for immunotherapy to treat infectious and noninfectious diseases of the heart. J Immunol Res.

